# Supplementary material for: Are carbon emissions trading and green financial instruments synergistic? -Comprehensive quantitative research based on content analysis
Source: PLoS One. 2024 Mar 7;19(3):e0298601. doi: 10.1371/journal.pone.0298601 (PMC10919601; doi:10.1371/journal.pone.0298601)
Supplement: S1 Data — (ZIP) [file pone.0298601.s001.zip › Data/地级市Z_F工作报告.zip!/╔╜╢1⁄2/╝├─╧╩╨/└┤╫╘╣1⁄2╓┌║┼í╛┬φ┐╦╩2╛▌═°í┐_2003.docx]

各位代表：

　　现在，我代表市人民政府向大会报告工作请予审议，并请各位政协委员和其他列席人员提出意见。

　　一、过去五年政府工作的回顾

　　过去的五年，在省委、省政府和市委的领导下，全市人民高举邓小平理论伟大旗帜，认真实践"三个代表"重要思想，紧紧围绕实现济南"五年大变样"的战略目标，大力实施科技、产业、制度、环境四个创新，全市综合经济实力显著增强，城市面貌发生重大变化，综合服务功能更加完善，省会现代化建设取得了重要的阶段性成果。

　　国民经济跃上了新台阶，经济总体实力显著增强。过去的五年，是济南发展史上最好的时期之一。2002年，全市实现国内生产总值1200亿元，年均增长12.9%；地方财政一般预算收入66.25亿元，年均增长17.3%；全社会固定资产投资404.7亿元，年均增长17.2%。尤为重要的是经济结构调整取得显著成效。产业结构发生重大变化，第一产业产业化程度提高，特色农业呈现良好发展态势；第二产业产业升级速度加快，支柱产业竞争能力明显增强；第三产业全面快速发展，新的产业业态迅速成长，三次产业的比重由11.6∶46.2∶42.2调整为8.2∶41.8∶50。所有制结构有所改善，形成了多种经济成份共同发展的新格局，非公有制经济增加值占国内生产总值的比重达到33%，提高了7个百分点。企业规模结构不断优化，规模以上工业企业实现销售收入、利税，分别是五年前的1.7倍和1.6倍；16户重点企业集团实现增加值占全市规模以上工业增加值的比重达到37.7%。技术结构得到提升，信息产业的加速和倍增作用增强，高新技术产业增加值占全市规模以上工业增加值的比重上升到23.16%。

　　各项改革深入发展，对外开放步伐加快。国有企业改革力度加大，现代企业制度建设稳步推进，企业的市场主体地位基本确立，投资主体多元化开始形成。对7大企业集团实行了国有资产授权经营，组建了6家国有资本营运机构，初步建立了国有资本营运监管体系。通过债转股，五年累计为企业减负31.2亿元，企业负债率下降，资产质量明显提高。顺利落实了农村第二轮土地承包制度，全面实施了农村税费改革。要素市场得到长足发展，技术市场基本形成，劳动力、人才市场成为人力资源配置的主要载体，住房市场进一步发育，资金市场不断成熟，2002年全市存贷款余额均超过2000亿元。整顿和规范市场经济秩序取得阶段性成果，企业经营和群众消费环境有了改善。积极稳妥地推进社会保障制度改革，全市城镇社会保险体系初步建立，社会救助体系不断完善，社区建设成效明显。新一轮政府机构改革顺利完成，政府职能进一步转变，清理取消审批项目617项，政府提速有了良好开端。大力实施经济国际化战略，外向型经济有了新的发展。五年累计实际利用外资19.2亿美元，占改革开放以来累计利用外资的53.8%；2002年全市完成出口总值(海关)7亿美元，年均增长6.24%；对外承包工程和劳务合作营业额达到9000万美元，年均增长48.3%；来我市投资的世界500强企业达到13家。

　　城市面貌发生了重大变化，承载能力和服务功能明显增强。五年来，城市建设累计投资595亿元，是上一个五年的3.5倍。人口城镇化率由35.4%提高到49.7%。城市交通设施建设加快。新建、改建了绕城高速等市区50多条主次干道和一批国道、省道干线公路、山区公路，完成了济南机场主跑道及联络道扩建工程，陆路、空运能力大大增强。城市各项配套设施建设取得重大进展。建成了总库容近1亿立方米的鹊山、玉清湖两座水库，完成了小清河济南段干流治理工程，配套完善了污水处理和垃圾无害化处理系统，城市用气普及率、热化率分别达到94%和38.2%。采取有效措施治理大气污染，2002年市区空气质量二级以上天数比1998年增加173天。泉城特色建设迈出新步伐。对三大名胜进行了扩建整治，抢救修复了一批被占压、填埋的名泉。泉城旅游品牌不断提升，年接待游客突破千万人次大关。大规模开展了城市绿化工程建设，城市绿地覆盖率和人均公共绿地面积分别达到37%和7.8平方米。数字化城市建设取得可喜成果，信息化基础设施达到全国先进水平。县城和小城镇建设也取得了重大进展。

　　各项社会事业全面发展，城市文化品味不断提高。科技投入增加，创新步伐加快，科技三项经费年均增长30%，取得重要科技成果1000多项，专利申请总量14821件，高新技术开发区作为科技创新"龙头"和载体的作用日益明显。各级各类教育发展加快，市区基本普及高中阶段教育，普通高考升学率由五年前的46%提高到79.9%，全市社会力量办学院校从308所发展到442所，全社会终身教育体系逐步形成。文化事业不断繁荣，涌现出一大批优秀剧目，获得了539项省和国家级奖励，文物保护与考古发掘取得了重大进展。群众体育跨入全国先进行列，竞技体育先后获省级以上比赛金牌438枚。社区卫生服务保持全国领先水平，新型卫生监督体系已经形成。计划生育工作取得明显成效，人口自然增长率连续五年控制在5‰以内，人口素质进一步提高，全市平均预期寿命达到74.3岁。统计、审计、技术监督、安全监督、工商行政管理等执法监督部门认真履行了职能。新闻出版、广播电视、社科研究、外事侨务、民族宗教、妇女儿童、老龄、残疾人等事业都取得了新成绩。

　　城乡居民收入有较大增长，生活质量有了新的提高。2002年城镇居民人均可支配收入达到8982元、农民人均纯收入3356元，分别比1997年增长61.2%和29.1%。城乡居民储蓄存款643.9亿元，比1997年翻了一番。食品供应丰富，质量安全水平提高。城市恩格尔系数从41.8%下降到32.9%，农村恩格尔系数从51.2%下降到39.8%。平均每百户城市居民家庭拥有电话95.3部、移动电话71.9部、家用电脑32.4台。城市居民人均住宅使用面积17.7平方米，农村居民人均生活用房面积30.6平方米，分别比1997年增加2.3平方米和3.5平方米。五年实现城镇就业、再就业18万人，城镇登记失业率控制在4%以内，城镇居民最低生活保障金由120元提高到208元，保障范围扩大，基本实现了应保尽保。农村扶贫开发取得了新的进展。

　　民主法制建设深入推进，精神文明建设结出了丰硕成果。坚持依法治市，切实推进政府法制建设，依法行政水平不断提高，法律服务体系日益健全。普法教育继续深入，公民法律意识明显增强。廉政建设稳步发展，反腐败斗争步步深入。社会治安综合治理富有成效，社会秩序稳定。群众性精神文明创建活动广泛开展，涌现出一批体现时代精神、在全国具有一定影响的先进典型。国防教育更加扎实，民兵预备役工作、人民防空建设取得新成绩。我市先后获得国家园林城市、全国优秀旅游城市、全国科技工作先进城市、全国民族团结进步模范市、全国社会治安综合治理优秀城市、全国双拥模范城等荣誉称号。

　　这些成绩的取得，是在省委、省政府和市委的正确领导下，在人大、政协的监督支持下，全市人民努力奋斗的结果，是驻济各界人士共同参与的结果。在此，我代表市人民政府，向付出辛勤劳动的全体市民、人大代表、政协委员、各民主党派、工商联、各人民团体、驻济人民解放军和武警官兵、港澳台同胞、海外侨胞以及为济南建设和发展作出贡献的同志们、朋友们表示崇高的敬意和衷心的感谢！

　　各位代表，在肯定成绩的同时，我们也要清醒地看到发展中还存在一些突出问题。主要是：市场机制在资源配置中的基础性作用依然不强，制度和机制配套改革滞后于经济和社会的发展。经济结构性矛盾比较突出，传统产业改造升级任务很重，国有经济比重仍然较大，民营经济的发展步伐不快。经济国际化水平较低，外贸出口能力较弱，投资环境亟待改善。区域经济发展不平衡，农业和农村经济基础薄弱。部分城市居民和农民收入增长缓慢，就业和社会保障的压力很大，有些群众生活还比较困难。城市的规划、建设和管理层次有待提高，经营城市的路子需要进一步拓宽。一些政府部门的法制观念和服务观念不强，行政成本高、办事效率低、官僚主义等现象还不同程度地存在。这些问题有待于在今后的工作中采取更加务实有效的措施加以解决。

　　二、今后五年发展总的指导思想和主要奋斗目标

　　今后五年，是济南现代化建设的关键时期，也是可以大有作为的重要机遇期。党的十六大提出了全面建设小康社会的奋斗目标，充分表达了全国人民的迫切愿望。根据党的十六大精神，结合我市的实际，市委七届十次全会提出了全面建设小康社会，提前基本实现现代化的奋斗目标。新一届政府必须肩负起这一重大历史使命，在省委、省政府和市委的领导下，在人大、政协的监督支持下，带领全市人民经过顽强拼搏，使济南的经济和社会发展跃上新的台阶。

　　我们应当清醒地看到，济南未来的发展是在国内外激烈竞争的大趋势下进行的。在党的十六大精神指引下，全国各地区特别是大城市、中心城市，纷纷调整发展战略，争创新的发展优势，竞争的广度和深度都上升到新的层面。面对这一新的形势，我们必须跟上时代，加快发展。济南作为沿海经济大省的省会城市，也完全具备加快发展的条件：经过改革开放以来特别是实施"五年大变样"以来的建设，我市已经具备了相当的经济实力、较好的产业基础，并且形成了一批具有活力的新兴产业；省委、省政府对济南的发展极为关心并寄予厚望，省直机关、驻济单位和部队始终如一地关心、支持和参与济南建设，这一得天独厚的条件是我市加快发展极为有利的因素；全市人民加快现代化建设的愿望越来越迫切，把济南建设得更加美好，已成为全市人民的强烈追求和一致行动。我们要以更大的气魄和决心，解放思想，抓住机遇，乘势而上，加快发展，为建设"大而强、富而美"的新山东做出新的更大的贡献。

　　今后五年发展总的指导思想是：高举邓小平理论伟大旗帜，全面贯彻"三个代表"重要思想，认真落实党的十六大提出的各项任务，紧紧围绕全面建设小康社会、率先基本实现现代化的目标，以增强城市综合竞争力、争创发展新优势为主攻方向，大力实施科技、产业、制度、环境四个创新，加快推进信息化、市场化、国际化、城市化和新型工业化进程，在培植主导产业、壮大县域经济、发展外向型经济、建设现代化城市、深化市场取向改革上实现重大突破，为把济南建设成为经济繁荣、文化发达、功能完善、生态良好、富裕文明的现代化中心城市而奋斗。

　　今后五年经济和社会发展的主要奋斗目标是：

　　国民经济实现新的跨越。社会主义市场经济体制进一步完善，较好地形成反映经济规律、符合国际惯例的经济管理体制和运行机制；国内生产总值年均增长12%，到2007年达到2100亿元，提前实现第一个翻番；地方财政一般预算收入年均增长13%；全社会固定资产投资年均增长13%；社会消费品零售总额年均增长11%；出口创汇年均增长16%；实际利用外资年均增长12%。力争各项主要经济指标在全国同类城市中位次前移。经济市场化、国际化和结构高新化进程加快，构筑起现代产业体系的基本框架，三次产业的比重达到6∶44∶50。以信息化带动工业化，以工业化促进信息化，走新型工业化道路，形成以高新技术产业为先导、基础产业和制造业为支撑的新格局。传统服务业实现升级改造，现代服务业得到充分发展，城市综合服务功能和聚集辐射能力明显增强。农业现代化进程进一步加快，农民组织化程度、农业产业化经营程度和农产品质量安全水平大幅提高，生态农业、设施农业、创汇农业和观光农业得到更快发展，实现农业持续增效和农民稳定增收。

　　城市现代化建设取得重大突破。按照"新区开发、老城提升、两翼展开、整体推进"的总体思路，构筑新世纪现代化中心城市的发展框架。坚持高起点规划、高质量建设、高效能管理、高水平经营的方针，进一步拓展发展空间，完善城市功能，改善城市环境，突出泉城特色，全面提升城市的现代化水平。枢纽型、功能型城市基础设施体系更加完善，中心城区建设品位明显提升，人口、交通、生态压力得到缓解。历史文化名城保护、泉城特色标志区和城市风貌带建设上升到新的水平，城市人居和发展环境质量有明显改善。建立起高效规范的城市管理模式，完善市场化的城市经营机制。加快数字化城市建设步伐，进一步提高城市化水平，人口城镇化率达到58%。力争把4县(市)城区建成规划科学、规模适度、环境优美的现代化中小城市。

　　各项社会事业全面繁荣。初步建成现代国民教育体系，形成全省领先的学习型城市。构建起以高等院校院所为依托的知识创新体系、以企业为主体的技术创新体系和以中介机构为载体的科技服务体系，科技进步对经济、社会的推动作用更加明显。不断完善文化创新体系，建设文化强市迈出新步伐。全民健身和医疗卫生体系更加完善，全民健康水平明显提高。人口年均自然增长率控制在5‰以内。

　　人民生活水平和质量明显提高。建立起促进就业再就业的长效机制，五年实现就业再就业25万人，城镇登记失业率控制在4%以内。城镇居民人均可支配收入年均增长7%，农民人均纯收入年均增长6%。社会保障制度进一步完善，城镇社会养老保险实现各类经济组织及社会人员的全面覆盖。城市居民人均住宅使用面积和农村居民人均生活用房面积分别达到19.3平方米和33平方米。城镇人均公共绿地面积8.8平方米。居民的精神文化消费层次有较大提升。

　　民主法制和精神文明建设开创新局面。民主法制建设取得新的进展，各级政府的依法行政水平有较大提高，各类行政执法活动得到有效规范，全民法制观念明显增强，法律服务、法律援助工作进一步加强。完善村民自治和城市居民自治机制，有效推进基层民主政治建设，全体市民的综合文明素质进一步提高。

　　实现上述目标，任务光荣而艰巨，只要我们统一思想，抓住机遇，奋力拼搏，真抓实干，就一定能够夺取全市改革与发展的更大成绩。在工作的指导和把握上要做到六个坚持：一是坚持以经济建设为中心。把加快发展作为富民强市的第一要务，遵循经济发展规律和市场法则，更新观念，大胆实践，勇于创新，积极探索加快发展的新途径。二是坚持向改革开放要动力、要活力。坚持"有进有退"、"有所为有所不为"的方针，搞好经济布局的战略调整，完善创新经济和社会快速发展的体制和机制。实施国际化战略，围绕实现两个市场的加速融合和两种资源的优化配置，努力构筑全方位、宽领域、纵深化对外开放的新格局。三是坚持实行重点突破。围绕培植新的经济增长点、壮大经济实力，突出重点工作，抓住关键环节，集中人财物力，强化综合措施，力求实现大的突破。四是坚持社会主义物质文明、政治文明和精神文明协调发展。在抓好物质文明建设的同时，大力加强政治文明和精神文明建设，努力为改革与发展创造良好的环境，充分发挥政治文明、精神文明对物质文明的支撑和促进作用。五是坚持正确处理改革、发展、稳定的关系。把改革的力度、发展的速度和社会可承受的程度统一起来，在社会稳定中推进改革发展，通过改革发展促进社会稳定。六是坚持强化省会意识。广泛动员和凝聚方方面面的力量，形成共同参与省会城市建设的强大合力。

　　三、全面做好开局之年的工作

　　要全面实现今后五年的奋斗目标，开局之年至关重要。今年全市经济社会发展的主要预期目标是：国内生产总值增长12%；地方财政一般预算收入增长13.71%；社会消费品零售总额增长12%；外贸出口增长18%，实际利用外资增长20%；全社会固定资产投资增长20%；城镇居民人均可支配收入增长8%，农民人均纯收入增长5%；城镇登记失业率控制在4.5%左右；人口自然增长率控制在5‰以内。

　　(一)以培植富有竞争力的主导产业为重点，加快新型工业化发展步伐。加快现代化建设步伐，基础在工业。要进一步强化"工业强市"思想，牢固树立大抓工业、抓大工业的强烈意识，努力提高工业在三次产业中的比重，逐步走出一条科技含量高、经济效益好、资源消耗低、环境污染少、人力资源优势得到充分发挥的新型工业化路子。加大经济结构调整力度，在培植壮大主导产业上狠下功夫。进一步创造条件扶持软件、信息家电等重点企业做大做强。抓好一批企业的信息化试点工作，用信息技术再造企业生产管理流程。积极发展对经济增长有突破带动作用的高新技术产业，提高生物医药、新材料和环保等产业的产业化水平，大力建设特色高新技术基地、高新技术密集带，努力把齐鲁软件园建成全国一流的软件基地。采用高新技术和先进适用技术改造提升传统产业，大力振兴装备制造业，努力提高装备工业的智能化水平和加工制造能力；做强纺织服装、医药化工、冶金建材等行业。以扩大骨干企业队伍为重点，对工业资源进行战略性整合。加快重点企业集团的膨胀发展，对销售收入、利税前100名的工业企业大户，强化政府协调和跟踪服务，推动企业加快发展步伐。加大对中小企业的扶持力度，建立健全面向中小企业的社会服务体系，扶持中小企业向"专、精、特、新"方向发展。鼓励和支持加快技术创新步伐，加大技改投入力度，力争工业项目投资超过130亿元。大力实施名牌战略，依托优势企业，着力打造有影响力的名牌产品。把园区建设作为培强做大工业经济的重要载体和亮点，高新技术开发区要结合东部产业带规划的实施实现二次创业；经济开发区等园区和重点集团工业园要加快建设步伐。各县(市)区要从本地实际出发，把办好工业园区作为农村工业化与城镇化的关键环节，落实好招商引资和项目建设责任制，走好城乡一体、整体推进的路子，进一步开创城市工业和县乡工业协调发展的新局面。企业的发展，归根到底在人才。要从战略高度，加强企业家队伍的培育和建设，推行国有企业经理人员公开招聘和选拔制度，完善国有资产保值增值考核办法，建立科学合理的经营者业绩考核体系，鼓励和支持对企业经营者和技术骨干实行年薪制、期权制等激励办法。

　　(二)以加快推进农业和农村经济结构调整为重点，努力实现农业增效、农民增收、农村繁荣。把建设现代农业、发展农村经济，作为全面建设小康社会、率先基本实现现代化的重点来抓。优化农业和农村产业结构，进一步调整粮经种植比例，使经济作物比重有较大提升。高度重视养殖业在农民增收中的重要作用，扩大养殖业的规模，提高养殖业的质量与效益。提高农业产业化经营水平，抓好食品深加工等拉长农业产业链的重点环节，提高农产品附加值。加大对龙头企业扶持力度，着力培育农村经济合作组织和种植业、养殖业、加工业大户，积极发展一村一品的特色经济。加快建立健全与国际接轨的农产品质量标准体系、检验检测体系和认证体系，抓好标准化基地建设，提高农业标准化水平。多渠道开拓农产品出口营销市场，提高农产品国际化水平，增加农产品创汇能力。进一步健全完善农业科技创新和推广服务体系，健全农产品市场信息体系和农业支持保护体系，提高农业可持续发展能力。抓好农村交通、电力、通讯等基础设施建设。加强农田基本建设和水利建设，推广发展节水型农业和生态农业，搞好荒山、荒滩、荒地、荒坡的合理开发与利用，提高防汛抗旱能力。实施保泉绿化、平原绿化、封山育林和绿色通道工程。切实落实好党在农村的基本政策，尊重农民的市场主体地位和自主经营权。积极稳妥地推进土地使用权的合理流转，不断提高土地的规模化、集约化水平。把农村经济发展与城镇化建设结合起来，大力发展农村二、三产业，加快农村富余劳动力转移。巩固农村税费改革成果，做好开发式扶贫工作，切实减轻农民负担。

　　(三)以扩大规模和提升层次为重点，大力发展现代服务业。把拓展现代服务业作为提升经济结构、增加社会消费、增强城市综合服务功能、提升城市现代化服务水平的重点来抓。发展新型商贸业态，搞好大型连锁经营，提高专业批发市场水平，扩大市场交易规模，提高市场辐射能力。对现有物流资源进行有效整合，从基地建设、要素配置和区域性联合方面，按市场化要求进行规范运作，特别要在大型第三方物流企业的培育和发展上取得较大突破。发挥好驻济金融机构融资能力强的优势，为金融发展创造条件、搞好服务，增强金融对经济的支撑作用。大力发展信息服务业，进一步拓展电子商务在各领域的运用。按照"开发大市场、形成大产业"的思路，切实做好人文历史、山泉湖河的文章，进一步打造三大名胜精品品牌，加大旅游市场主体的培育力度，抓好特色旅游产品的开发和推介，力争旅游总收入突破90亿元。改造提升餐饮服务业，发展美食文化。继续加快房地产业发展，增强信贷支持能力，不断满足城镇居民日益增长的住房消费需求。拓展物业服务领域，加强物业管理，进一步提高物业服务质量。积极发展会展业，扩展领域，膨胀规模，扩大影响，增加效益。鼓励咨询、广告、认证、评估等中介机构的发展，更好地为经济和社会发展服务。

　　(四)以经营城市为重点，进一步搞好城市建设和管理。高起点搞好城市功能分区规划，完善专业规划和具体实施方案，增强规划的科学性、前瞻性、指导性。要加强城市规划的执法监督，放开规划设计市场，重大规划面向国内外公开招标。老城改造在保护好历史文化名城、突出泉城特色的基础上，坚持统一规划、连片开发、配套完善、综合整治的方针，成熟一片，启动一片。高质量建设好发挥基础作用的重点工程，集中力量搞好燕山立交、高架路北延、机场二期、火车站广场改造等在建工程。要强化新开工项目的前期准备工作，认真实施好泉城特色标志区一期工程、东部产业带综合建设工程、西部经济开发区起步工程、街巷综合整治工程。要加强工程项目的统一组织领导，进一步建立健全项目法人制等制度。高效能搞好城市管理，不断强化"两级政府、三级管理、四级网络"的管理体制，建立市场取向的城市管理机制，大力推进标准化管理，实施城市管理综合执法，不断提高城市管理效能。加强社区建设，充分发挥社区在城市管理中的基础作用。高水平搞好城市经营，政府依法垄断土地一级市场，放开搞活二级市场，坚持土地规划、征用、开发、供应、管理"五统一"，实现城市土地资源效能的最大化；积极推进城市供水、供暖、供气、公交等基础设施的市场化和社会化，盘活有形资产，增加经营效益；多渠道筹措和增加建设资金，推动城市建设走自求平衡、滚动发展、良性循环的路子。

　　(五)以体制和机制创新为重点，不断完善社会主义市场经济体制。按照市场经济的要求，积极引导国有资本合理流动，加快国有资本从竞争性领域退出的步伐。以产权制度改革为核心，逐步建立权利、义务、责任相统一，管人、管事、管资产相结合的国有资产管理体制，提高国有资产的运行效能。加快国有企业的优化重组和结构调整，进一步提高企业竞争力。要认真研究国有企业改革中的重点和难点问题，切实实现资产变现、债务处理、职工安置等方面的新突破。推进事业单位的改革，按照政事分开和市场化原则，实行人员聘用制，加快推进经营开发类事业单位转制的步伐，逐步做到股权多元化、经营市场化、管理企业化。深化分配制度改革，鼓励和支持劳动、资本、技术、管理等生产要素按贡献参与分配。整顿规范市场经济秩序，依法严厉打击制售假冒伪劣商品、偷税骗税和价格欺诈等不法行为。健全安全生产监督管理体系，抓好安全生产专项整治。进一步加大产权、劳动力、技术等要素市场建设和市场化价格机制改革的力度。

　　(六)以扩大利用外资为重点，开创外经外贸工作新局面。在更大范围、更广领域、更高层次上参与国内外两个市场的竞争与合作，力争外贸出口和实际利用外资的增长幅度超过全省平均水平。把利用外资放在首位，精心筛选招商项目，精心组织招商工作，努力提高招商的成功率。重点抓好关联度大、技术水平高的大项目和对世界500强、世界知名大企业的招商工作。不断扩大招商领域，加大国内社会和民间资金的招商引资工作力度，努力形成新一轮招商引资热潮。努力扩大出口，壮大出口企业队伍，优化出口产品结构，在继续抓好传统产品出口的同时，着力提高机电和高新技术产品的出口比重。切实抓好加工贸易，发展好协作配套型项目，延伸加工贸易生产链条，形成大进大出新格局。大力开拓出口市场，争取更多的产品出口。积极实施"走出去"战略，鼓励有实力的企业境外兴业。扩大境外工程承包和劳务合作的规模，增强创汇能力。进一步改善和优化投资软环境，严格岗位督查和部门监察制度，努力营造一个稳定透明的政策和法律环境，诚信公平的市场环境，廉洁高效的管理环境，安心舒心的社会环境。

　　(七)以改组、改制和园区建设为重点，推动民营经济快速发展。民营经济是社会主义市场经济的重要组成部分，是加快我市经济发展的潜力所在。要努力创造促进民营经济发展的良好环境和浓厚氛围，使民营企业创业人员社会上有地位、政治上有荣誉、经济上有实利，以促进民营经济更快更好地发展。鼓励和支持国有集体企业改制一批、骨干民营企业膨胀一批、市外民营企业引进一批、广泛发动发展一批，不断壮大民营企业队伍。加强对民营企业的引导和服务，创造条件，鼓励和支持有实力、有条件的民营企业参与国有企业的战略性重组，实现投资主体的多元化；对符合国家法律法规、产业政策以及加入世贸组织后对外资放开的领域，放手让个体私营企业生产经营。鼓励和引导民间资本投向能源、交通、供水、环境保护等基础设施及公用事业建设。要积极推动民营经济园区向特色园区、专业园区和科技园区发展；引导与支持民营企业加快制度创新、结构创新、技术创新。努力形成多种经济成份优势互补、互相促进、共同发展的局面。

　　(八)以扩大就业和实现"两个确保"为重点，完善社会保障体系。就业是民生之本，一定要全力抓好。实施积极的就业政策，立足发展经济解决就业问题，大力发展就业容量大的劳动密集型产业，发展各种非公有制经济，创造更多的就业岗位。贯彻市场就业的方针，广开就业门路，推行灵活就业方式，促进劳动者自谋职业。努力抓好下岗失业人员的再就业，完善劳动力中介服务网络，搞好职业培训和上岗培训，提高下岗失业人员的就业能力。健全街道(镇)和社区劳动保障服务工作机构，开展对特困就业群体的就业援助。高度重视、关心弱势群体，加强社会救助工作。完善社会保障制度，进一步提高社会保障能力，特别要加大社会保险的征缴、扩面和清欠工作，社会劳动保险要逐步实现各类经济组织及从业人员的全面覆盖，将外商投资企业、私营企业、个体工商业户和自由职业者以及外来经商务工人员全部纳入基本养老保险范围。

　　(九)以适应人们不断增长的物质和精神需求为重点，加快推进各项社会事业。大力推进科技进步，建设知识产权保护体系，集成优化科技资源，加紧人力资源能力建设，努力构筑科技人才高地。发展完善技术市场，发挥科技中介机构作用，推进产学研紧密结合。抓好技术攻关，提高科技成果转化率。发挥好高新技术风险投资公司、科技型中小企业技术创新基金、民营企业科技投资担保基金的作用，加速培育新的经济增长点和科技创新点。抓好科普工作，提高市民的科学知识水平。大力拓展优质教育资源，积极推进和深化素质教育，全面提高教育质量。继续巩固"普九"成果，加快高中阶段教育的发展。大力促进民办教育发展，鼓励多种模式办学。拓宽职业教育、成人教育面，提高全民受教育水平。整合文化资源，发展文化产业，繁荣文化创作，加强基层文化建设，依法保护、合理开发利用文物资源，提高城市文化品味。加强卫生队伍建设，健全社区卫生体系，提高预防保健和基本医疗服务水平，确保无重大传染疾病发生。深入开展全民健身活动，提高竞技体育水平。积极开展计划生育优质服务，搞好综合改革、政策推动和综合治理，稳定低生育水平，促进优生优育。做好外事侨务、民族宗教、妇女儿童、残疾人等工作，推动新闻出版、广播电视、社科研究等各项社会事业的发展。

　　(十)以依法治市和以德治市为重点，切实加强民主法制和精神文明建设。深入开展普法教育，提高全民法律意识，大力推进基层民主法制建设，创新基层政权建设体制。积极推进依法治市、依法行政的进程，理顺行政执法体制，落实行政执法责任，强化行政执法监督，切实增强政府工作人员，特别是领导干部和执法人员依法行政的自觉性。坚持政府向人大及其常委会报告工作、向人民政协通报情况的制度，自觉接受监督。认真办好人大代表议案、建议和政协提案。充分发挥各民主党派、工商联、无党派人士参政议政作用，多方听取人民群众意见和要求。积极支持工会、共青团、妇联等群众团体开展工作。继续加强社会治安综合治理，强化群防群治工作力度，严厉打击严重刑事犯罪活动，提高社会整体防范能力。加强基层矛盾纠纷调处和群众来信来访工作，预防和化解各类不安定因素。深入进行党的十六大精神和"三个代表"重要思想的宣传教育，认真落实《公民道德建设实施纲要》，深入开展创建文明社区、文明行业、文明村镇和争做文明市民活动，提高市民和城市的文明水平。继续做好拥军优属工作，认真落实优抚安置政策，积极支持国防建设，保持全国"双拥模范城"荣誉，努力争取新的发展。

　　四、转变政府职能，建设务实高效的人民政府

　　各级政府要身体力行"三个代表"重要思想，切实转变职能，推进行政管理体制创新，不断提高依法管理经济和社会事务的能力。

　　(一)解放思想，强化责任。要坚持党的解放思想、实事求是、与时俱进的思想路线，树立强烈的改革意识、创新意识和市场意识。积极探索改革新思路、发展新办法、工作新举措，努力解放和发展社会生产力。要强化政府责任、部门责任、领导责任，建立健全责任考评机制和责任追究机制，做工作，办事情，都要切实做到任务明确，责任到位，严格考核。各级政府一定要有强烈的历史使命感和时代责任感，以实现济南新一轮的发展为根本职责，忠于职守，努力工作，决不辜负时代与人民的重托。

　　(二)转变职能，提高效率。按照建立服务型政府的要求，切实改变管理经济和社会事务的方式，深化行政体制改革，努力克服体制性障碍，继续推进政企分开、政事分开和政社分开，把政府职能转移到经济调节、市场监督、社会管理和公共服务上来，努力营造公平的经济发展环境。坚持政务公开，保障群众的知情权、参与权和监督权；精减审批项目，简化审批程序，提高行政效率，降低行政成本，逐步形成行为规范、运转协调、办事公开、务实高效的行政管理体制，推动政府工作全面提速。要加强各职能部门为企业服务的协调配合，营造安全高效的网上服务系统，为企业提供全过程服务。建立完善重大行政决策合法性、可行性论证机制，建立决策、执行、监督体系，提高行政决策科学化、民主化水平；把建设"诚信政府"作为政府建设的一项重要目标，不断提高政府的公信力。

　　(三)廉洁奉公，执政为民。各级政府工作人员要进一步增强公仆意识，正确对待和使用好手中权力。认真落实领导干部、公务员廉洁从政的各项规定，建立和完善思想教育、制度规范、监督制约、查案惩处、干部保护、廉政责任六个机制，提高拒腐防变能力。深入开展反腐败斗争，依法重点查办领导干部违法违纪、执法人员贪赃枉法等案件。严格监管土地批租、工程招标、产权交易等环节，严格领导干部经济责任审计和"收支两条线"管理，严肃财经纪律，从源头上防止腐败行为的发生。坚持勤俭办一切事业，珍惜民资民力，精打细算，严禁铺张浪费，切实把有限的资金用在提高群众生活和增强经济发展后劲上。

　　(四)扎实工作，转变作风。大力弘扬求真务实、讲求实效的优良作风，力戒官僚主义、形式主义。坚持一切从实际出发，从我们正在做的事情出发，着眼于解决发展中存在的实际问题，倡导真抓实干、雷厉风行的工作作风。坚持实践第一的观点，勇于实践，敢于创新，从实践中获取正确认识指导实践。要深入基层，调查研究，体察民情，了解民意，以满腔热忱和高度责任感去解决人民群众关心的热点难点问题。采取切实有力的措施，解决好城市困难职工、农村困难群众的生产生活问题。各级政府务必把作风建设作为建设务实高效的人民政府的一件大事切实抓好。

　　各位代表，党的十六大描绘了新世纪、新阶段全面建设小康社会、加快推进社会主义现代化建设的宏伟蓝图。我们要紧密团结在以胡锦涛同志为总书记的党中央周围，在省委、省政府和市委的领导下，高举邓小平理论伟大旗帜，全面实践"三个代表"重要思想，解放思想，团结奋斗，开拓创新，扎实工作，为全面建设小康社会、率先基本实现现代化而努力奋斗！
